# Reconstitution of the phosphodiesterase 6 maturation process important for photoreceptor cell function

**DOI:** 10.1016/j.jbc.2023.105576

**Published:** 2023-12-16

**Authors:** Sneha Singh, Dhiraj Srivastava, Kimberly Boyd, Nikolai O. Artemyev

**Affiliations:** 1 Department of Molecular Physiology and Biophysics, The University of Iowa Carver College of Medicine, Iowa City, Iowa, USA; 2 Department of Ophthalmology and Visual Sciences, The University of Iowa Carver College of Medicine, Iowa City, Iowa, USA

**Keywords:** photoreceptor, phosphodiesterase 6, phototransduction, chaperone, HSP90, protein folding

## Abstract

The sixth family phosphodiesterases (PDE6) are principal effector enzymes of the phototransduction cascade in rods and cones. Maturation of nascent PDE6 protein into a functional enzyme relies on a coordinated action of ubiquitous chaperone HSP90, its specialized cochaperone aryl hydrocarbon receptor-interacting protein-like 1 (AIPL1), and the regulatory Pγ-subunit of PDE6. Deficits in PDE6 maturation and function underlie severe visual disorders and blindness. Here, to elucidate the roles of HSP90, AIPL1, and Pγ in the maturation process, we developed the heterologous expression system of human cone PDE6C in insect cells allowing characterization of the purified enzyme. We demonstrate that in the absence of Pγ, HSP90, and AIPL1 convert the inactive and aggregating PDE6C species into dimeric PDE6C that is predominantly misassembled. Nonetheless, a small fraction of PDE6C is properly assembled and fully functional. From the analysis of mutant mice that lack both rod Pγ and PDE6C, we conclude that, in contrast to the cone enzyme, no maturation of rod PDE6AB occurs in the absence of Pγ. Co-expression of PDE6C with AIPL1 and Pγ in insect cells leads to a fully mature enzyme that is equivalent to retinal PDE6. Lastly, using immature PDE6C and purified chaperone components, we reconstituted the process of the client maturation *in vitro*. Based on this analysis we propose a scheme for the PDE6 maturation process.

Among 11 families of type I cyclic nucleotide phosphodiesterases (PDE), members of the sixth family (PDE6) are specialized as the principal effector enzymes in the vertebrate phototransduction cascade in rods and cones (1, 2, 3). Distinctive features of PDE6 include exclusive expression in photoreceptor cells, regulation by small inhibitory γ-subunits (Pγ), and an exceptionally high catalytic activity following G-protein mediated disinhibition (2). Rod PDE6 is composed of a catalytic PDE6AB heterodimer and two copies of Pγ_r_, whereas cone PDE6 is a homodimer of catalytic PDE6C subunits each associated with the cone-specific Pγ_c_ subunit. Prior to its trafficking to the signaling outer segment compartment, newly synthetized PDE6 proteins are assembled and maturated in the house-keeping inner segment of photoreceptors. In contrast to other PDE enzymes, and possibly to enable some of its features, rod and cone PDE6 require the complex of HSP90 with a specialized cochaperone aryl hydrocarbon receptor (AhR)-interacting protein-like 1 (AIPL1), for its folding and maturation (4, 5, 6). Failure of PDE6 to properly maturate into a functional enzyme caused by mutations in AIPL1 results in type 4 Leber congenital amaurosis (LCA4), a severe early-onset form of blindness in children (7). Consistent with AIPL1 serving as a critical cochaperone, its knockout (KO) in mice leads to a drastic reduction of the PDE6 protein level and no enzymatic activity, followed by severe and rapid degeneration of the retina (8). Furthermore, it has been suggested that AIPL1 influences the proper assembly and stability of PDE6 through its interaction with the PDE6A subunit (9). In addition to AIPL1, the Pγ subunit also plays a critical role in PDE6 maturation. Deletion of Pγ_r_ (encoded by the *Pde6g* gene) in mice leads to rapid retinal degeneration (10). Importantly, the PDE6AB catalytic dimer was reported to be present in a native-like conformation in the *Pde6g* KO retina prior to retinal degeneration, yet its cGMP hydrolytic activity was markedly impaired, suggesting that Pγ_r_ is necessary for expression of PDE6AB activity *in vivo* (10). The cochaperone-like role of Pγ_c_ was supported by the heterologous expression of PDE6C in HEK293T cells, whereby only a small fraction of cGMP-hydrolytic activity of PDE6C is obtained following the enzyme co-expression with AIPL1 alone compared to when it is co-expressed with AIPL1 and Pγ (11).

To further explore the roles of AIPL1 and Pγ in the maturation of PDE6, we established the heterologous expression of PDE6C in insect Sf9 cells allowing purification and characterization of the enzyme. We show that co-expression with AIPL1 allows conversion of the readily aggregating inactive PDE6C species into the PDE6C dimers, a small fraction of which is fully catalytically active. Reconstitution of this preparation of PDEC with Pγ did not increase the fraction of active enzyme. However, co-expression of PDE6C with AIPL1 and Pγ resulted in the entire population of PDE6C molecules being equivalent to native PDE6 from mouse retina. To determine if the contribution of Pγ to the maturation of PDE6AB parallels that in the maturation of PDE6C, we examined PDE6AB activity in mouse retinas lacking Pγ_r_ and PDE6C. Surprisingly, the residual rate of cGMP hydrolysis in the mutant mouse retinas at P10 (prior to photoreceptor degeneration) and at P30 (complete photoreceptor degeneration) was not different, suggesting that no PDE6 activity is present at P10. Thus, the requirement of Pγ for maturation of rod PDE6AB, unlike that of cone PDE6C, is absolute. To delineate the steps in PDE6 maturation, we reconstituted the process using immature PDE6C and the key chaperone components *in vitro*. Our analysis suggests that AIPL1 and Pγ interact with the loaded HSP90/PDE6C complex to induce conformational changes leading to client maturation. Recent cryo-EM studies advanced our understanding of the HSP90/client loading and maturation processes involving co-chaperones structurally related to AIPL1, such as AhR interacting protein (AIP/XAP2) and FKBP51/52 (12, 13, 14, 15, 16). We propose a scheme of PDE6 maturation that incorporates our results into the context of these studies.

## Results

### Expression of PDE6C in insect cells in the absence or presence of AIPL1

For expression of human PDE6C, Sf9 cells were infected with baculovirus encoding the EGFP- fusion protein of human PDE6C or co-infected with two baculoviruses, one of which also encoded the His_6_-tagged mouse AIPL1. The standard purification procedure involved immunoaffinity chromatography over EGFP-nanobody resin followed by PDE6C release with HRV-3C protease and size-exclusion chromatography (SEC) using the Superose 6 column. PDE6C expressed in the absence of AIPL1 (endogenous HSP90 is present) lacked cGMP hydrolytic activity and eluted as a very broad peak corresponding to MW of ∼300 to 1000 kDa (Fig. S1). An SDS-PAGE analysis of the SEC fractions indicated the approximately equimolar presence of a ∼100 kDa PDE6C band and a co-migrating 70 kDa protein band. The latter protein was identified by mass spectrometry to be a *Spodoptera frugiperda* HSP70 (Fig. S2).

**Figure 1:**
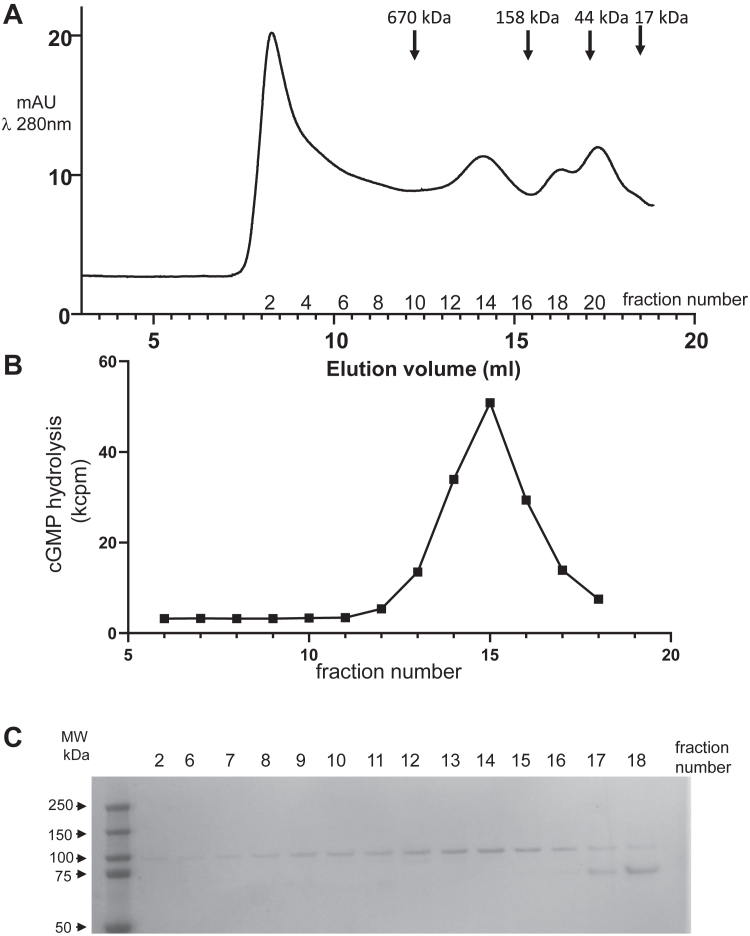
**Expression of PDE6C in insect cells in the presence of AIPL1.** Extract from Sf9 cells transfected with baculoviruses encoding EGFP-PDE6C and AIPL1 was adsorbed onto EGFP-nanobody resin. PDE6C released with HRV-3C protease was preincubated with 3 mM ATP and subjected to SEC chromatography on a Superose 6 column. SEC elution (*A*) and cGMP-hydrolytic activity (*B*) profiles of PDE6C. *C*, Coomassie-stained gel after SDS-PAGE of the SEC fractions. Note that the peak activity (fraction 15) has shifted from the PDE6C protein peak (fraction 14).

When PDE6C was co-expressed with AIPL1, the peak of PDE6C protein eluted with an apparent MW of ∼300 to 350 kDa, and it contained substoichiometric amounts of HSP70, indicating partial separation of the proteins (Fig. S3). This PDE6C protein hydrolyzed cGMP with the peak activity corresponding to the protein peak (Fig. S3). Considering the ATPase cycle of HSP70 and the fact that the ADP-bound HSP70 binds substrates tightly and releases them in the ATP-bound form, we examined the effect of addition of ATP to the PDE6C sample eluted from EGFP-nanobody resin with HRV-3C protease. The SEC elution profile of the ATP-treated sample of PDE6C showed a distinct peak with a retention volume similar to that without added ATP and a virtually complete separation from HSP70 during SEC (Fig. 1). However, the peak PDE6 activity for this sample has shifted from the protein peak by 0.5 ml and corresponded to MW of ∼250 kDa (Fig. 1).

### Properties of the PDE6C enzyme expressed in the presence of AIPL1 and the absence of Pγ

**Figure 2:**
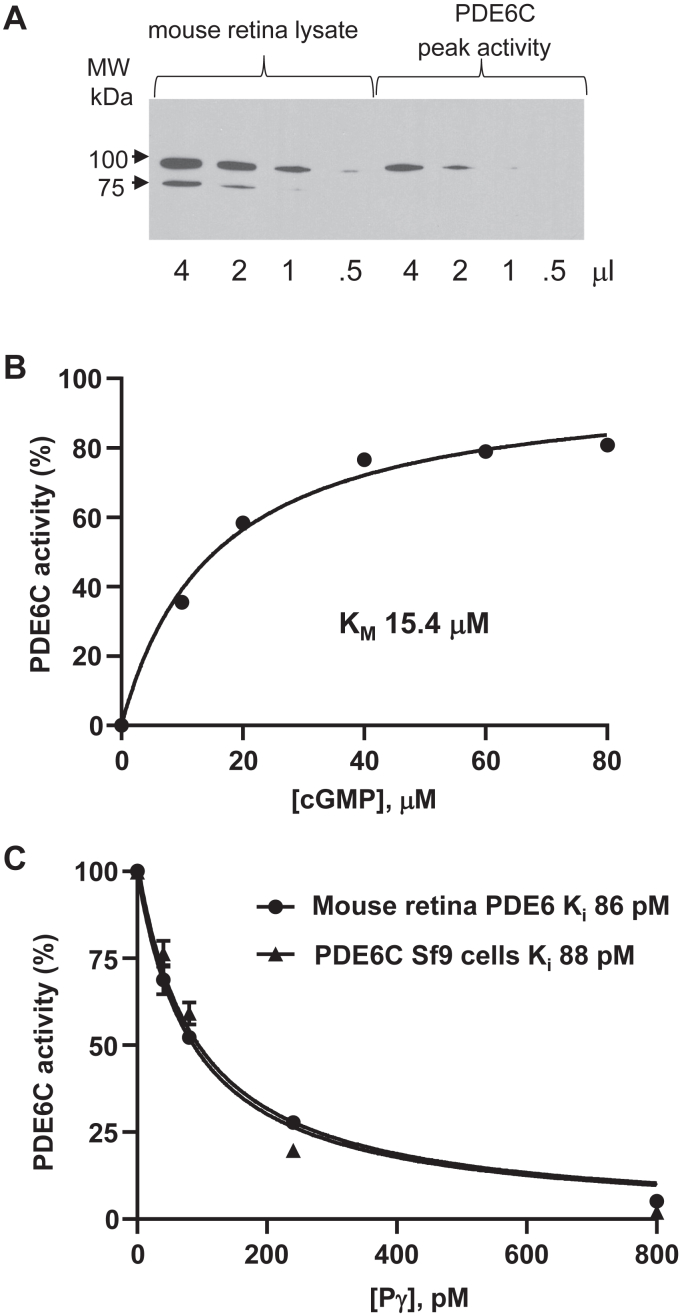
**Properties of the PDE6C enzyme expressed in the presence of AIPL1.***A*, Western blot analysis for normalization of PDE6AB protein content in C57BL/J mouse retina lysates and PDE6C protein content in the peak PDE6 activity fraction 15 (Fig. 1*B*) using PDE6 antibodies (18). *B*, Michaelis-Menten analysis of PDE6C. Representative experiment is shown. From three similar experiments (Mean ± SD), K_M_ = 13.2 ± 2.0 μM cGMP. *C*, comparison of inhibition of PDE6C and trypsin-activated PDE6AB from mouse retina extract by Pγ_r_. Representative experiment is shown. From three similar experiments (Mean ± SD), K_i_ = 95 ± 7 pM for PDE6C; K_i_ = 89 ± 8 pM PDE6AB.

To assess the specific activity of the recombinant PDE6C protein in the peak activity fraction relative to that of trypsin-activated PDE6 in mouse retina (17), we measured the rates of cGMP hydrolysis in the samples normalized for the amounts of PDE6 proteins. The maximal catalytic activities of mature human cone PDE6 and mouse rod PDE6 are expected to be similar (18). The Western blotting was performed with antibodies that recognize cone and rod PDE6 equally well (18). The matching levels of cGMP hydrolysis were achieved for the mouse retina extract and PDE6 in the peak activity fraction with 1:2000 and 1:200 dilutions, respectively. From the Western blotting, the retina extract contained ∼3-fold higher PDE6 protein than the heterologously expressed PDE6C (Fig. 2*A*). Accordingly, the normalized activity of PDE6C was ∼30 to 35% of that of trypsin-activated PDE6AB in mouse retina. As the PDE6 activity in the peak protein fraction (# 14) is ∼1.5 fold lower and the PDE6 protein level ∼2-fold higher compared to the peak activity fraction (#15) (Fig. 1, *B* and *C*), the specific activity of PDE6C in the peak protein fraction can be estimated to be 10 to 12% of mouse PDE6AB.

Since PDE6C co-expressed with AIPL1 was catalytically active, we were able to examine the key properties of the enzyme, *e.g.*, K_M_ for cGMP hydrolysis and K_i_ for inhibition by Pγ. The K_M_ value for the purified PDE6C was calculated to be 15 μM (Fig. 2*B*). This value matches closely the K_M_ estimated previously for native bovine PDE6C (19), and it is similar to the K_M_ estimates for rod PDE6 (20). Our analyses of the inhibition of the recombinant PDE6C by purified recombinant Pγ_r_ revealed the K_i_ value of 88 pM, which was equivalent to the K_i_ for Pγ_r_ inhibition of trypsin-activated PDE6 from mouse retina (Fig. 2*C*). Thus, we concluded that only a relatively small fraction of PDE6C co-expressed with AILPL1 is properly assembled, but this fraction is functionally equivalent to native trypsin-activated cone and rod PDE6 enzymes.

Considering the evidence for the cochaperone role of Pγ in the maturation of PDE6 (10, 11), we tested the ability of exogenously added Pγ to increase the fraction of functional PDE6C in our preparations. Following the addition of Pγ to purified PDE6C and incubation, PDE6C activity was measured after Pγ was removed with trypsin treatment. The resulting PDE6C activity was not different from that in control samples without the addition of Pγ. Thus, Pγ does not appear to act subsequently to PDE6 maturation in the presence of AIPL1, but it rather acts as a PDE6 cochaperone during the maturation process.

### Co-expression of PDE6C with AIPL1 and Pγ leads to a mature enzyme

**Figure 3:**
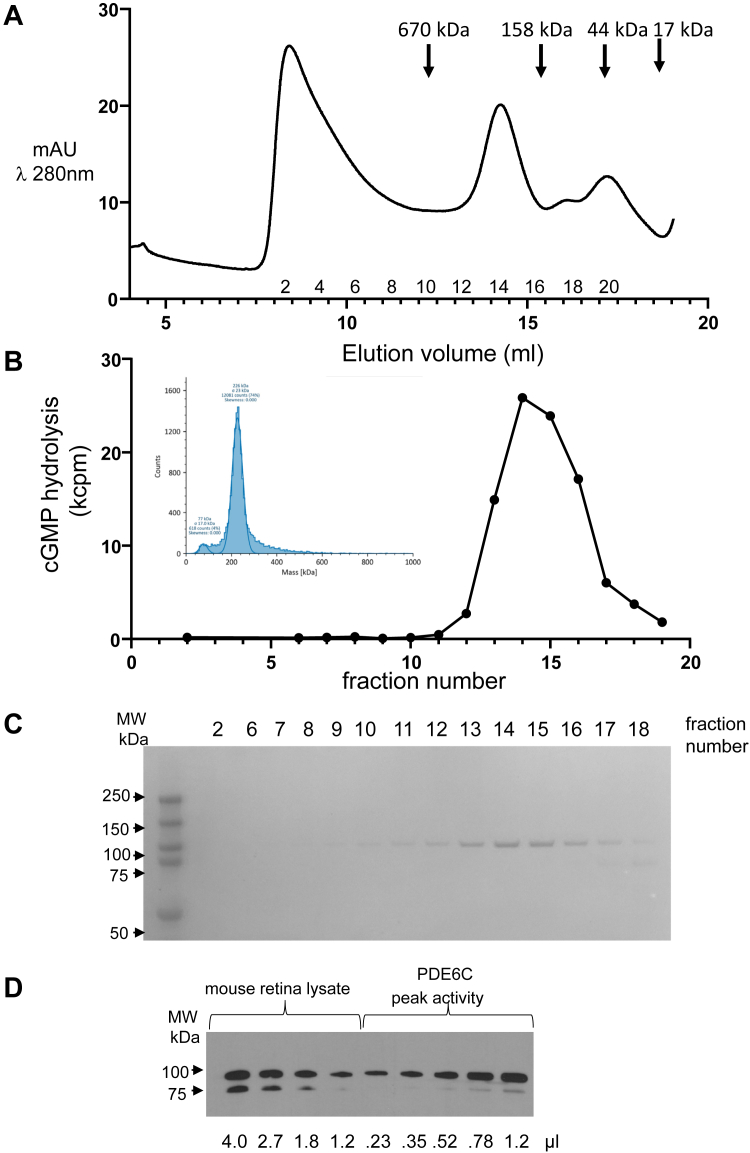
**Expression of PDE6C in insect cells in the presence of AIPL1 and Pγ.** Extract from Sf9 cells transfected with baculoviruses encoding EGFP-PDE6C, AIPL1 and Pγ was adsorbed onto EGFP-nanobody resin. PDE6C released with HRV-3C protease was preincubated with ATP and subjected to SEC chromatography on a Superose 6 column. SEC elution (*A*) and cGMP-hydrolytic activity (*B*) profiles of PDE6C. Inset: analysis of the PDE6C in the peak SEC fraction by mass photometry. *C*, Coomassie-stained gel after SDS-PAGE of the SEC fractions. Note that the peak of PDE6C activity corresponds to the PDE6C protein peak. *D*, Western blot analysis for normalization of PDE6AB protein content in C57BL/J mouse retina lysates and PDE6C protein content in the peak PDE6 fraction using PDE6 antibodies.

As the addition of Pγ had no effect on PDE6C activity following its expression in the presence of AIPL1, we co-expressed PDE6C with AIPL1 and Pγ using infection of Sf9 cells with the three corresponding baculoviruses. The elution profile indicated a well-resolved PDE6C protein peak corresponding to MW of ∼250 kDa (Fig. 3*A*). Analysis of the concentrated peak fraction by mass photometry reveals a largely monodispersed species of 226 kDa (Fig. 3*B*, inset), which is consistent with the predicted MW of the PDE6C dimer complexed with two copies of the Pγ subunit. The peak of PDE6 activity matched the protein peak (Fig. 3, *B* and *C*). The peak fractions 14 to 16 were combined and concentrated to determine the specific activity of PDE6C. To compare specific activities of mouse retina PDE6AB and PDE6C expressed in the presence of AIPL1 and Pγ, the rates of cGMP hydrolysis were measured in the normalized samples treated with trypsin to degrade Pγ. The matching levels of cGMP hydrolysis were attained for the mouse retina extract and in the concentrated PDE6C sample with dilutions 1:2000 and 1:10,000, respectively. Based on the Western blotting analysis, the retina extract contained ∼4.5-fold less PDE6 protein than the PDE6 SEC fraction (Fig. 3*D*). Thus, we estimate the specific activity of PDE6C in this preparation at ∼110% of mouse PDE6AB activity. Considering a degree of uncertainty as to whether the maximal activities of native cone and rod PDE6 enzymes are equal, this estimate suggests that the recombinant PDE6C is fully mature. Using an extinction coefficient of ε_280_ = 117,690 for PDE6C/Pγ to determine the concentration of PDE6C catalytic subunits in the PDEC sample, we estimated the rate of cGMP hydrolysis by a single catalytic site at ∼400 mol cGMP/s. Since the assays were conducted at 25 °C and [cGMP]< K_M_, the k_cat_ value for the PDE6C subunit would easily exceed 1000/s under physiological conditions.

### Pγ is absolutely required for the maturation of rod PDE6

**Figure 4:**
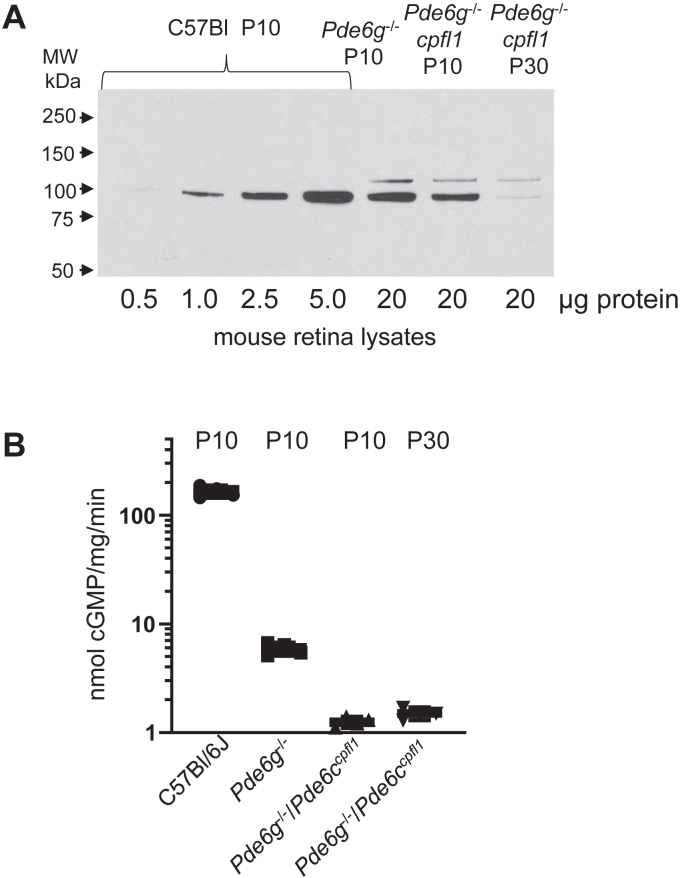
**PDE6 protein and activity levels in mutant mice.***A*, relative PDE6 protein levels in *Pde6g*^−/−^, *Pde6g*^−/−^/*Pde6c*^*cpfl1*^, and C57BL/J mice (control) at P10 (for *Pde6g*^−/−^/*Pde6c*^*cpfl1*^ also at P30) were assessed by Western blotting of retina lysates using PDE6 antibodies. *B*, the rates of cGMP hydrolysis were measured in trypsin-treated retina lysates of *Pde6g*^−/−^, *Pde6g*^−/−^/*Pde6c*^*cpfl1*^, and C57BL/J mice (control) at P10 and for *Pde6g*^−/−^/*Pde6c*^*cpfl1*^ also at P30. The PDE6 activity values are (nmol cGMP hydrolyzed•mg^−1^•min^−1^): C57BL/J, 165.5 ± 19.4 (n = 4); *Pde6g*^−/−^, 5.9 ± 0.8 (n = 4); 1.3 ± 0.2 (n = 4); 1.5 ± 0.2 (n = 3) (Mean ± SD).

The capacity of a small fraction of cone PDE6C to properly maturate in the absence of Pγ raised the question of whether the same is true for the rod PDE6. Our efforts to express functional PDE6AB in a heterologous system by co-expression with AIPL1 in the absence or the presence of Pγ have been unsuccessful. Accordingly, to answer the above question we turned to a mouse model. The original Pγ_r_ knockout mouse model was not appropriate for the purpose, because its retina contains functional cone PDE6C prior to degeneration (10). Therefore, we generated mice lacking both Pγ_r_ and PDE6C by breeding *Pde6g*^−/−^ and *cpfl1* mice (21, 22). The relative PDE6 protein levels were assessed by Western blotting and the rates of cGMP hydrolysis were measured in trypsin-treated retina lysates of *Pde6g*^−/−^, *Pde6g*^−/−^/*Pde6c*^*cpfl1*^, and C57BL/J mice (control) at P10 prior to retina degeneration. The protein levels of PDE6AB in mutant *Pde6g*^−/−^ and *Pde6g*^−/−^/*Pde6c*^*cpfl1*^ mice were similar and reduced by ∼5-fold compared to that in control mice (Fig. 4*A*). This observation is consistent with the earlier analysis of the *Pde6g*^−/−^ mouse (10). The rate of cGMP hydrolysis was reduced by ∼25-fold in *Pde6g*^−/−^ retinas and ∼130-fold in *Pde6g*^−/−^/*Pde6c*^*cpfl1*^ retinas, indicating that cone PDE6C accounts for ∼3 to 3.5% of PDE6 activity in mouse retina at P10 (Fig. 4*B*). To determine if the residual <1% of cGMP hydrolysis in *Pde6g*^−/−^/*Pde6c*^*cpfl1*^ mice can be attributed to rod PDE6AB, we also measured the rate of cGMP hydrolysis in *Pde6g*^−/−^/*Pde6c*^*cpfl1*^ retina lysates at P30 when photoreceptor cells completely degenerate. Surprisingly, the levels of cGMP hydrolysis in the mutant mice at P10 and P30 were similar, suggesting that the low activity in *Pde6g*^−/−^/*Pde6c*^*cpfl1*^ retina originates from PDEs other than the photoreceptor PDE6 enzymes (Fig. 4*B*). Thus, PDE6AB expressed in mouse retina in the absence of Pγ_r_ is completely inactive.

### Reconstitution of PDE6C maturation *in vitro*

**Figure 5:**
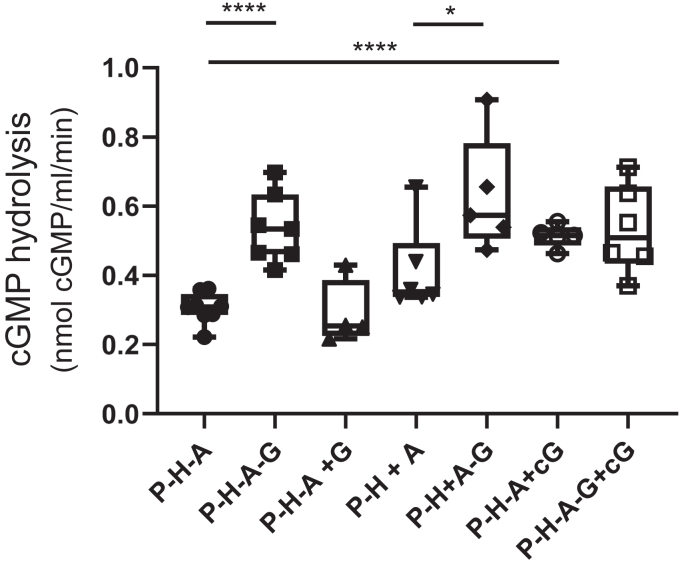
**Reconstitution of PDE6C maturation *in vitro*.** Immature EGFP-PDE6C adsorbed to EGFP-nanobody resin (P) was reconstituted with HSP90 (H), AIPL1 (A) and Pγ (G) as described in Experimental procedures. cGMP-hydrolysis by PDE6C was measured following the following reconstitution protocols: P-H-A, PDE6C was preincubated with HSP90 and AIPL1; P-H-A-G, PDE6C was preincubated with HSP90, AIPL1, and Pγ; P-H-A+G, PDE6C was preincubated with HSP90 and AIPL1 followed by addition of and incubation with Pγ; P-H+A, PDE6C was preincubated with HSP90 followed by addition of and incubation with AIPL1; P-H+A-G, PDE6C was preincubated with HSP90 followed by addition of AIPL1 and Pγ. The PDE6 activity values are (nmol cGMP hydrolyzed•ml resin^−1^•min^−1^): P-H-A, 0.30 ± 0.04 (n = 8); P-H-A-G, 0.54 ± 0.10 (n = 7); P-H-A+G, 0.29 ± 0.10 (n = 4); P-H+A, 0.41 ± 0.12 (n = 6); P-H+A-G, 0.63 ± 0.17 (n = 5); P-H-A+cG, 0.51 ± 0.03 (n = 5); P-H-A-G+cG, 0.53 ± 0.13 (n = 6). One-way ANOVA, *p*∗∗∗∗ = 0.0001. Unpaired *t* test P-H-A *versus* P-H-A-G, *p*∗∗∗∗ < 0.0001; P-H-A *versus* P-H-A+cG, *p*∗∗∗∗ < 0.0001; P-H+A *versus* P-H+A-G, *p*∗ = 0.035 (Mean ± SD).

To investigate if the maturation of PDE6C can be reconstituted *in vitro*, we expressed immature EGFP-PDE6C in the absence of AIPL1 and Pγ, and we used the protein attached to EGFP-nanobody resin as the client in the reconstitution protocol. This preparation of PDE6C has no PDE6 activity, and it also contains HSP70 (Figs. S1 and S2). Following incubation with purified chaperone components and trypsin treatment to remove Pγ, we measured the ability of PDE6C to hydrolyze cGMP as a readout of the enzyme maturation. Incubation of PDE6C with HSP90 and AIPL1 induced measurable PDE6C activity (Fig. 5). Pγ enhanced PDE6 maturation *in vitro* when PDE6C was incubated with HSP90, AIPL1, and Pγ all together by about 2-fold (Fig. 5). Importantly, Pγ had no significant effect on the enzyme maturation when PDE6C was first incubated with HSP90 and AIPL1, followed by the addition of and incubation with Pγ (Fig. 5). Positive effects of Pγ on PDE6C maturation were also observed when the client was first incubated with HSP90 followed by addition of AIPL1 and Pγ (Fig. 5). Although the effects of Pγ under these experimental conditions were moderate, they indicate that Pγ acts on the chaperone-client complex and not subsequently to the client release from the complex.

Recent evidence suggests that cGMP bound at the noncatalytic sites of the GAFa domains is important for the folding and/or stability of rod PDE6 (23). We tested the potential role of cGMP-binding to the GAFa domain in folding of PDE6C. cGMP was added to the reactions at the concentration of 2 mM that would not be reduced significantly due to hydrolysis by maturated PDE6C. In the reconstitution reaction with PDE6C, HSP90, and AIPL1, cGMP enhanced PDE6 maturation comparably to the effect of Pγ (Fig. 5). However, the addition of cGMP to the reaction in the presence of Pγ had no significant effect, indicating that the effects of Pγ and cGMP on PDE6C maturation *in vitro* are not additive (Fig. 5).

### Molecular dynamics simulations to mimic immature PDE6

The dimerization interface of PDE6 catalytic subunits buries large hydrophobic surfaces, which would be prone to aggregation if the catalytic dimers are not assembled (Fig. S4). The propensity of PDE6C expressed in the absence of AIPL1 to aggregate suggests the failure of the PDE6 catalytic subunits to dimerize. We conducted MD simulations of the individual PDE6A and PDE6B subunits to gain insight into potential conformations of the catalytic subunits leading to their inability to assemble dimeric proteins. The rationale for these simulations is based on the assumption that the individual PDE6 domains, the GAFa-GAFb domains, and the catalytic domain, can fold independently of the HSP90/AIPL1 complex. This is true for at least the GAF domains, as the structure of the bacterially expressed cone PDE6 GAFa-GAFb construct reveals proper assembly and dimerization (24). Although there is no PDE6-specific evidence to support the assumption that the catalytic domains of PDE6 are largely folded prior to the protein maturation, the catalytic domain of related PDE5 had been expressed as a functional monomer in bacteria (25). Moreover, the AIP(XAP2)-dependent HSP90 client AhR is threaded through the HSP90 lumen as a monomeric protein prior to its heterodimerization with ARNT (14, 15). Thus, the PDE6 catalytic subunits are likely threaded through the HSP90 lumen as monomers with pre-folded GAF domains, and the use of monomers in simulations is justified. For the starting conformations of PDE6A and PDE6B, each subunit was extracted from the cryo-EM structure of native rod PDE6 (26). Multiple 50 to 200 ns simulation runs were conducted, and they revealed common features present in all MD trajectories. Consistent with the native-like dimeric structure of the cone PDE6C GAFa-GAFb construct (24), the GAF domains and the helical segment linking GAFa and GAFb domains (LH1) retained conformations similar to those in the starting structures (Fig. S5). However, the helical segment LH2 linking GAFb with the catalytic domain became kinked or twisted to the extent that would severely impede the ability of the PDE6 subunits to dimerize (Fig. S5). Upon dimerization of the kinked monomers, the PDE6 catalytic domains would clash with each other or with the GAFb domains of the partner subunit, although some conformations may allow partial dimerization of the GAF regions (Fig. S6).

## Discussion

**Figure 6:**
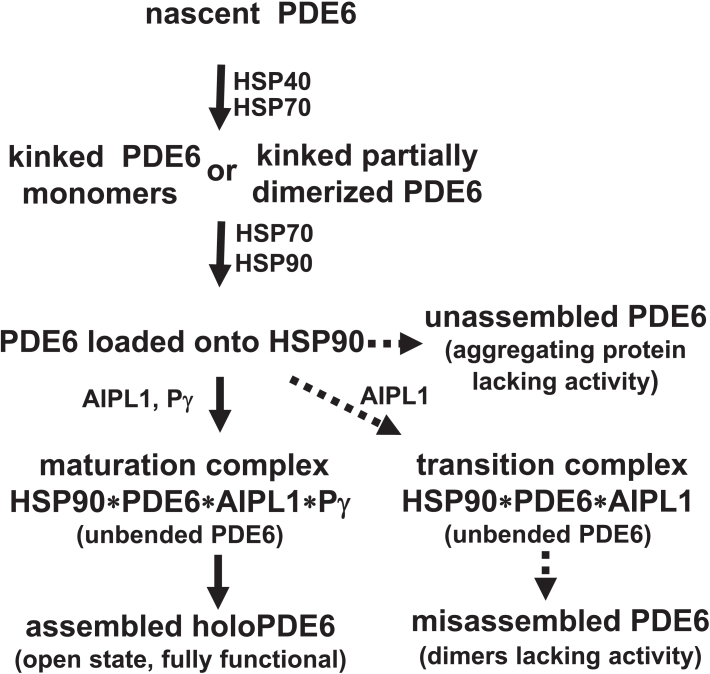
**Proposed scheme of maturation of PDE6 enzymes.** Individual domains, GAFa, GAFb, and possibly the catalytic domain, in newly synthesized PDE6A, PDE6B, or PDE6C subunits are folded by the HSP70/HSP40 machinery, but the dimerization interface is malformed or is partially formed at the GAFa-GAFb region, requiring HSP90 and cochaperones AIPL1 and Pγ for maturation. Crooked PDE6 catalytic monomers or partially dimerized PDE6 molecules are loaded onto HSP90 dimers with the assistance of HSP70 (12). When AIPL1 and Pγ interact with the loaded HSP90-PDE6 complex, they direct the formation of the full-length dimerization interface and correct “open” orientation of the catalytic domain. Upon ATP-hydrolysis by HSP90, PDE6 catalytic subunits are released from the chaperone complex and assemble mature holoPDE6. *Dashed arrows* indicate the paths in the absence of AIPL1 or Pγ.

Our analysis of heterologous expression of (PDE6)_2_Pγ_2_ in insect cells and reconstitution of the PDE6C maturation *in vitro* allows us to propose a scheme of PDE6 maturation (Fig. 6). Following the folding of nascent PDE6 by the HSP70/HSP40 machinery, PDE6 is loaded onto HSP90. The loading chaperone-client complex for PDE6 likely parallels that structurally described for the maturation of glucocorticoid receptor (GR) (12). The FKBP51/52-dependent client GR and the AIP(XAP2)-dependent HSP90 client AhR are loaded and threaded through the HSP90 lumen as monomeric proteins (12, 13, 14, 15), and we hypothesize that PDE6 is loaded as monomers too. However, since the GAFa-GAFb regions of PDE6C in the absence of the catalytic domain can properly fold and self-dimerize (24), it is possible that PDE6 is loaded onto HSP90 as a protein partially dimerized at the GAF-domain interface (Fig. 6). It is also possible that the chaperone machinery acts not only on nascent PDE6 but can also refold misassembled PDE6 dimers. HSP70 may help to unwind the misassembled PDE6C, which is then loaded onto HSP90 (12). AIPL1 and/or Pγ do not assist with the client loading but rather act at the later stages of the HSP90 cycle. In the absence of AIPL1, PDE6 catalytic subunits released from HSP90 fail to assemble into stable dimers and form aggregating complexes lacking PDE6 activity (Figs. 6 and S6). Binding of AIPL1 to the loaded HSP90/PDE6 complex and ATP-hydrolysis by HSP90 induces conformational changes in PDE6C allowing the catalytic subunits upon release to dimerize. According to our MD simulations, such conformational changes may involve unbending of the kinked PDE6 linker helix LH2, and LH2 is a likely candidate region to be threaded through the lumen of the HSP90 dimer. The unbending of LH2 in the absence of Pγ leads to PDE6 dimerization mostly into a misassembled state lacking catalytic activity. Misassembled PDE6 may resemble a “closed” state of PDE2 observed in the crystal structure, in which the catalytic pockets are occluded at the dimer interface (27).

This study provides particular insight into the role and mechanism of Pγ in the maturation of PDE6. We show that co-expression of PDE6C with AIPL1 and Pγ in insect cells leads to a fully mature PDE6C. However, Pγ does not cause any maturation of PDE6 in the absence of AIPL1 (11). Furthermore, the addition of Pγ to the mixture of misassembled and assembled PDE6C did not increase the yield of the functional enzyme. In the reconstitution experiments, Pγ had no effect when added after preincubation of immature PDE6C with HSP90 and AIPL1, but enhanced PDE6C maturation when added together with AIPL1 to HSP90 and the client. Accordingly, Pγ has no effect on the maturation of PDE6C once the protein is released from its complex with HSP90 and AIPL1. Apparently, Pγ interacts with the complex of HSP90 with PDE6 and AIPL1, and this directs the release of the catalytic subunits that form mature PDE6 heterotetramer (PDE6)_2_Pγ_2_ rather than misassembled PDE6 dimers (Fig. 6). Lowering the energy of the transition maturation complex may underline the catalytic effect of Pγ on PDE6 maturation. Possibly, Pγ facilitates rotation of the PDE6 catalytic domain required for the closed-to-open state change (26). The nature of the Pγ interaction with the maturation complex remains unclear, but it may involve Pγ binding to PDE6 and/or AIPL1. We have previously shown that Pγ can interact with the TPR-domain of AIPL1 (28). In addition, we demonstrated that binding of cGMP to the GAFa domain of PDE6C enhances the enzyme maturation *in vitro*. The effects of Pγ and cGMP on PDE6C maturation *in vitro* were not additive. This could be due to a common underlying mechanism involving the stabilization of the GAFa domains (26). The role of noncatalytic cGMP in folding of cone PDE6 may not be as critical as that for rod PDE6 since cGMP binding to the cone GAFa is less tight (19, 29). Expression of rod PDE6A is virtually eliminated in a mouse model with a mutation in PDE6A GAFa that impedes cGMP binding (23).

If HSP90 and AIPL1 assist PDE6 folding in the absence of Pγ, the outcome is different for cone and rod PDE6. PDE6C largely forms misassembled inactive dimers with a small fraction of properly assembled fully functional dimers. However, rod PDE6 in the absence of Pγ is completely misassembled. Original characterization of PDE6AB in the Pγ_r_ KO mice indicated that the protein is dimeric (10), and we present an analysis of PDE6AB in the *Pde6g*^−/−^/*Pde6c*^*cpfl1*^ mice which demonstrates that the enzyme has no activity. Different dependencies of cone and rod PDE6 on Pγ for maturation may contribute to the differences in the phenotypes caused by the loss of Pγ_r_ and Pγ_c_. Recent studies suggest that even a very low expression level of functional PDE6 can rescue rod photoreceptors from degeneration (23). The absolute requirement of Pγ for maturation of rod PDE6 is consistent with rapid retina degeneration in *Pde6g*^−/−^ mice, which resembles that in *rd1* mice (10, 21, 30). In contrast, targeted deletion of the cone-specific *Pde6h* gene in mice revealed a normal overall retinal morphology and no defects in cone-rod-driven signaling (31). The lack of the phenotype in *Pde6h*^−/−^ mice was attributed to “leak” expression of Pγ_r_ in cones. However, this phenotype (or lack thereof) differs from that in human patients with incomplete achromatopsia due to the loss-of-function mutation in the *PDE6H* gene (32). These patients revealed a severe dysfunction of L and M cones with relatively preserved S cone function (32). The ability of a small fraction of PDE6C to properly maturate in the absence of Pγ should certainly be considered a contributing factor to this phenotype. If L and M cones in human achromatopsia patients do not express cone and rod Pγ, they would fail to signal, but they can escape cell death due to residual PDE6C activity. Overall, a better understanding of the mechanism of PDE6 maturation and the roles of AIPL1 and Pγ will help to design therapeutic strategies to treat visual disorders linked to mutations in the *AIPL1*, *PDE6G*, and *PDE6H* genes.

## Experimental procedures

### Plasmids/cloning

For the bacterial expression of human HSP90β (1–724) and mouse AIPL1 used in the reconstitution experiments, the genes were cloned into modified pET21a and pET28a-CPD vectors as described (4, 5). A synthetic gene for bovine Pγ_r_ (33) with a modified internal NcoI site was cloned into the NcoI and BamHI sites of the pET15b vector for bacterial expression of the His6-tagged protein. For the expression of human PDE6C in insect cells, a PDE6C construct with truncation of the C-terminal 28 residues (aa 1–830) was generated as these residues are not essential for PDE6 maturation (4). cDNA coding for GFP and PDE6C were spliced together with primers containing HRV-3C protease and FLAG tag, and the spliced DNA was cloned into the pFastBac HT A vector (Invitrogen) using Gibson assembly protocol (34). cDNA coding for the full-length mouse AIPL1 with the N-terminal His6 tag and the TEV cleavage site and the HA-tagged bovine Pγ_r_ were subcloned into the pFastBac HT A vector using Gibson assembly.

### Recombinant bacmid generation and transfection

The baculovirus generation was performed using the Bac-to-Bac system. The pFastBac constructs of PDE6C, AIPL1, and Pγ_r_ were transformed in the DH10Bac strain of *E.coli*. The recombinant bacmids were selected by blue/white colony screening. Sf9 cells were transfected with the isolated bacmid using ExpiFectamine Sf Transfection Reagent (ThermoFisher). The P0 viruses were further amplified to a titer of P3 or P4 and the virus stocks were stored at 4 °C. Sf9 cells were cultured for expression in Sf-900 II Serum Free Medium (Gibco) at 27 °C, 125 rpm. The cells were infected at a density of 2.5 × 10^6^ cells/ml with the P3 virus stock of AIPL1 and P4 of PDE6C and Pγ_r_ using multiplicities of infection (MOI) of 2 to 3. The cells were harvested 48 h post-infection at 1000*g* for 15 min.

### Protein purification

The harvested Sf9 cells were resuspended in the lysis buffer containing 50 mM Tris-HCl (pH 7.5), 200 mM NaCl, 1 mM TCEP, 2 mM MgCl_2_, 5% glycerol (buffer A) supplemented with 2 mM Phenylmethylsulfonyl Fluoride and cOmplete, EDTA-free Mini Protease inhibitor cocktail (Roche). The cells were lysed using sonication followed by centrifugation of the lysate at 20,000 rpm for 1 h at 4 °C. The soluble fraction was incubated with the EGFP nanobody resin (P&CF, University of Iowa) for 1 h at 4 °C, and the resin was washed with buffer A to remove non-specifically bound proteins. The resin with bound PDE6C was used further in the *in vitro* reconstitution experiments with the chaperone complex. For the purification of samples of PDE6C expressed alone or co-expressed with AIPL1 or with AIPL1 and Pγ_r_, PDE6C was eluted with HRV-3C cleavage using elution with buffer A. The eluted proteins were incubated with 3 mM ATP for 10 min at 25 °C to allow separation of PDE6C from HSP70 that was present in the sample. The samples were then subjected to size exclusion chromatography using Superose 6 10/300 GL (GE Healthcare) equilibrated with a buffer containing 10 mM HEPES (pH 7.5), 150 mM NaCl, 1 mM TCEP and 2 mM MgCl_2_. Bacterially expressed human HSP90β and mouse AIPL1 used for reconstitution with PDE6C were purified as described previously (5). Bovine Pγ_r_ was expressed and purified similarly as described previously (35).

### PDE6 activity assays

When co-expressed with Pγ, PDE6C samples were treated with 0.1 mg/ml TPCK-Trypsin (Sigma) on ice for 10 min to selectively degrade Pγ, after which trypsin was inhibited with the addition of 10-fold excess of soybean trypsin inhibitor (Sigma). For the assays, PDE6C samples were diluted 100 to 10,000 fold. The assays were conducted in 40 μl (final volume) of 20 mM Tris–HCl (pH 7.5) buffer containing 120 mM NaCl, 2 mM MgSO_4_, 1 mM BME, 0.1 U bacterial alkaline phosphatase, 10 μM [^3^H]cGMP (100,000 cpm) (PerkinElmer) for 10 to 20 min at 25 °C. The reaction was stopped by adding AG1-X2 cation exchange resin (0.5 ml of 20% bed volume suspension) (Bio-Rad). Samples were incubated for 6 min at 25 °C with occasional mixing and spun at 10,000*g* for 3 min 0.25 ml of the supernatant was removed for counting in a scintillation counter.

For assays of PDE6 activity in retina lysates, for each genotype or age, two mouse retinas were homogenized by sonication (two 5-s pulses) in 120 μl of 20 mM Tris-HCl buffer (pH 7.5) containing 120 mM NaCl, 1 mM MgSO_4_, and 1 mM BME. After brief centrifugation (20,000*g*, 2 min, 4 °C) to remove cell debris, retinal lysates (typically, 2–6 mg protein/ml) were treated with trypsin (100 μg/ml) for 10 to 15 min at 25 °C. Trypsin treatment was terminated by the addition of 10× soybean trypsin inhibitor (SBTI, Sigma) and incubation for 5 min at 25 °C, followed by centrifugation at 20,000*g* for 3 min at 4 °C. The final dilutions of trypsin-treated retinal lysates in the assays of PDE6 activity were 1:1000 to 1:6000, and the activity assays were carried out as above.

### Reconstitution of PDE6C maturation *in vitro*

PDE6C expressed in the absence of AIPL1 and Pγ and bound to EGFP nanobody resin (20 μl) was used for the reconstitution experiments using the proteins and reagents with the following concentrations: 15 μM HSP90β, 7.5 μM AIPL1, 10 nM rod Pγ, 2.5 mM ATP in buffer containing 50 mM Tris-HCl (pH 7.5), 150 mM NaCl, 1 mM TCEP, 5 mM MgCl_2_ and 5% glycerol. The reactions were incubated for 45 min at 25 °C for the reconstitution of the components in one step, *e.g.*, P-H-A and P-H-A-G in Figure 5. The P-H-A and P-H-A-G reactions were additionally performed in the presence of 2 mM cGMP (P-H-A+cG and P-H-A-G+cG in Fig. 5). For the sequential reactions (*e.g.*, P-H-A+G, P-H+A, and P-H+A-G in Fig. 5), AIPL1 and/or Pγ were successively added after 30 min of pre-incubation of PDE6C with HSP90 or HSP90 and AIPL1. Thereafter, the reactions were continued for another 15 min. Following the incubation, the resin was washed four times with the PDE6 assay buffer and assayed for the PDE6 activity as above.

### Mass photometry

Mass Photometry experiments were performed on a Refeyn TwoMP (Refeyn Ltd). No. 1.5, 24 mm × 50 mm microscope coverslips (Thorlabs Inc) were cleaned by serial rinsing with Milli-Q water and HPLC-grade isopropanol (Sigma Aldrich), on which a CultureWell gasket (Grace Bio-labs) was then placed. All measurements were performed at 25 °C in Dulbecco's phosphate-buffered saline (DPBS) without calcium and magnesium (Thermo Fisher). For each measurement, 15 μl of DPBS buffer was placed in the well for focusing, after which 3 to 5 μl of 100 nM protein was introduced and mixed. Movies were recorded for 60 s at 50 fps under standard settings. MP measurements were calibrated using protein standard mixture: β-Amylase (56, 112 and 224 kDa), and Thyroglobulin (670 kDa). From the calibration, the estimated error of the MP measurements was 0.2%. MP data were processed using DiscoverMP (Refeyn Ltd).

### Mass spectrometry (LC-MS/MS)

PAGE and in-gel trypsin digestion of the 70 kDa band were performed as described previously (5). Mass spectrometry data were collected using an Orbitrap Fusion Lumos mass spectrometer (Thermo Fisher Scientific) coupled to an Easy-nLC-1200System (Proxeon P/N LC1400). Typically, the autosampler is set to aspirate 4 μl (estimated 0.4 μg) of reconstituted digest and load the solution on a 2 cm C18 trap (Thermo Scientific, Catalog. No. 164535) coupled to waste and an analytical column through a microtee assembly (IDEX, P/N UH-752). The analytical column is a 50 cm Acclaim PepMap 100C18 HPLC Columns (Thermo Scientific, Catalog. No. 164570) which elutes through a nano-bore stainless steel emitter that remains in contact with a gold-coated electrode held at the ESI tip voltage (ca. 1900 V). Peptides are desalted on the trap using 16 μl mobile phase A in 4 min. The waste valve is then blocked, and a gradient flows at a rate of 0.3 μl/min through the column just described. Peptides were separated in line with the mass spectrometer using an 86 min gradient composed of linear and static segments wherein buffer A is 0.1% formic acid and B is 80% ACN, 0.1% Formic acid. The gradient first holds at 4% for 3 min then makes the following transitions (%B, min): (2, 0), (4, 3) (35, 63), (60, 73), (98, 76), (98, 86). Peak lists in the form of mgf files were submitted for the search using Protein Prospector ‘Batch-Tag Web’ (36).

### Generation of mice

Generation, genotyping, and the phenotype of the *Pde6g*^*CreERT2/CreERT2*^ mutant mice (referred to as *Pde6g*^−/−^) have been described previously (21, 30). B6.CXB1-Pde6c^*cpfl1*^/J mice were acquired from the Jackson Laboratory (22). *Pde6g*^−/−^/*Pde6c*^*cpfl1*^ homozygous for the loss-of-function mutations in the *Pde6g* and *Pde6c* genes were generated by breeding the mutant mice and genotyped by Transnetyx. All experimental procedures involving the use of mice were performed in accordance with the National Institutes of Health guidelines and the protocol approved by the University of Iowa Animal Care and Use Committee.

### Antibodies

Anti PDE6 antibodies (PDE6com) equally recognizing all PDE6 catalytic subunits were generated as described previously (18). Procedures for immunoblotting were performed as described previously (37).

### Molecular dynamics simulations

MD simulations were performed with YASARA Structure 18.2.7 using the md_runfast macro. For the starting conformations of PDE6A and PDE6B, each subunit was extracted from the cryo-EM structure of native rod PDE6 (PDB 6MZB) (26). The simulations were run in cuboid simulation cells with 30 Å extension of the cell on each side of the protein using the AMBER14 force field in water at a temperature of 310 K, pH of 7.4, and NaCl concentration of 0.9%. Particle mesh Ewald summation was used to compute long-range columbic interactions with a periodic cell boundary and a cutoff of 8 Å. Four independent MD runs were performed on each of the starting models with simulation times ranging from 50 to 200 ns. Analysis of MD trajectories for per residue fluctuation (RMSFs) was performed using the MD_analyzeres macro and by specifying superposed Cα atoms within the macro.

### Statistical analyses

Unless otherwise indicated, measurements were taken from at least three independent experiments, and data are shown as mean value and standard deviation. Measurements between two groups were compared using *t* test. The differences among multiple groups were analyzed with one-way ANOVA and Tukey follow-up test. GraphPad Prism 10 (GraphPad Software Inc) was used for data fitting and analysis.

## Data availability

Other data supporting the study findings are available from the corresponding author upon reasonable request.

## Supporting information

This article contains supporting information.

## Conflict of interest

The authors declare no conflicts of interest.
