# Supplementary material for: Reconstitution of the phosphodiesterase 6 maturation process important for photoreceptor cell function
Source: J Biol Chem. 2023 Dec 16;300(1):105576. doi: 10.1016/j.jbc.2023.105576 (PMC10819763; doi:10.1016/j.jbc.2023.105576)
Supplement: Supplemental Figures S1–S6 [file mmc1.pdf]

A

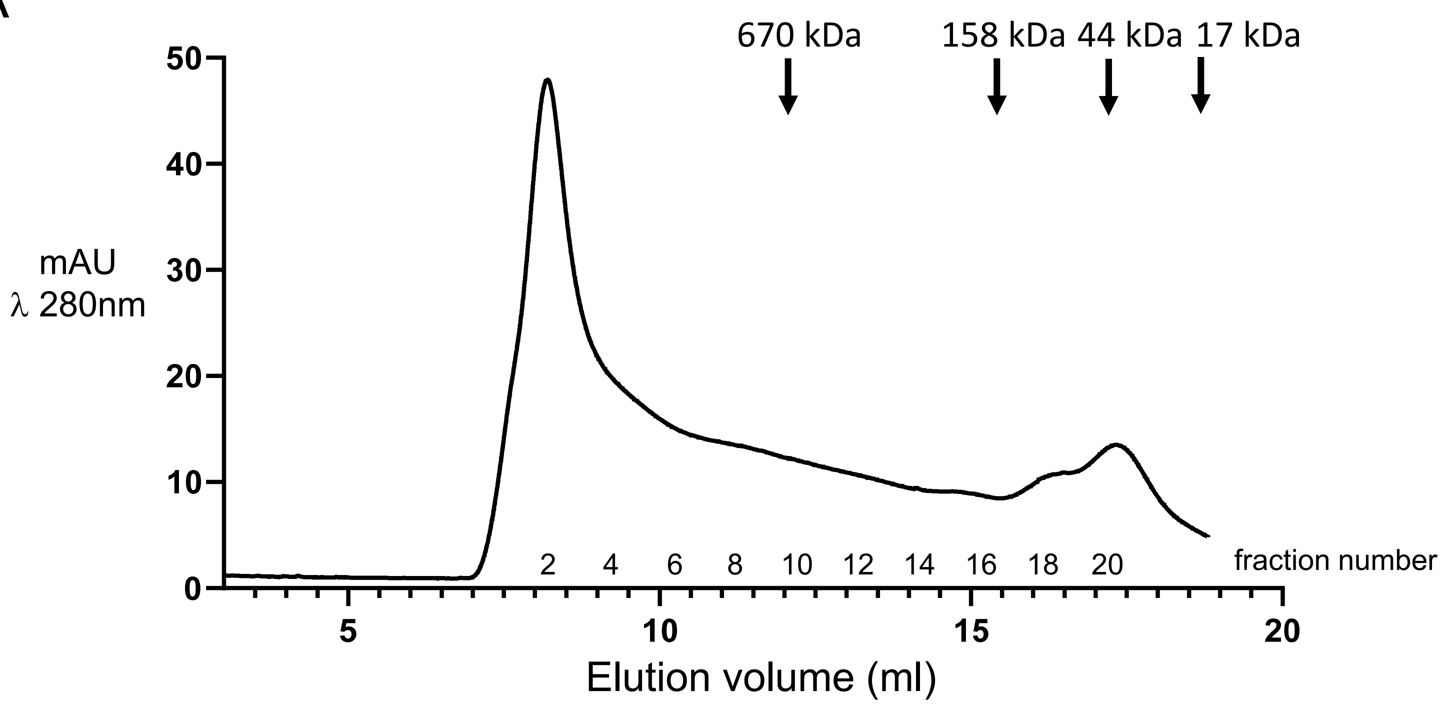

B

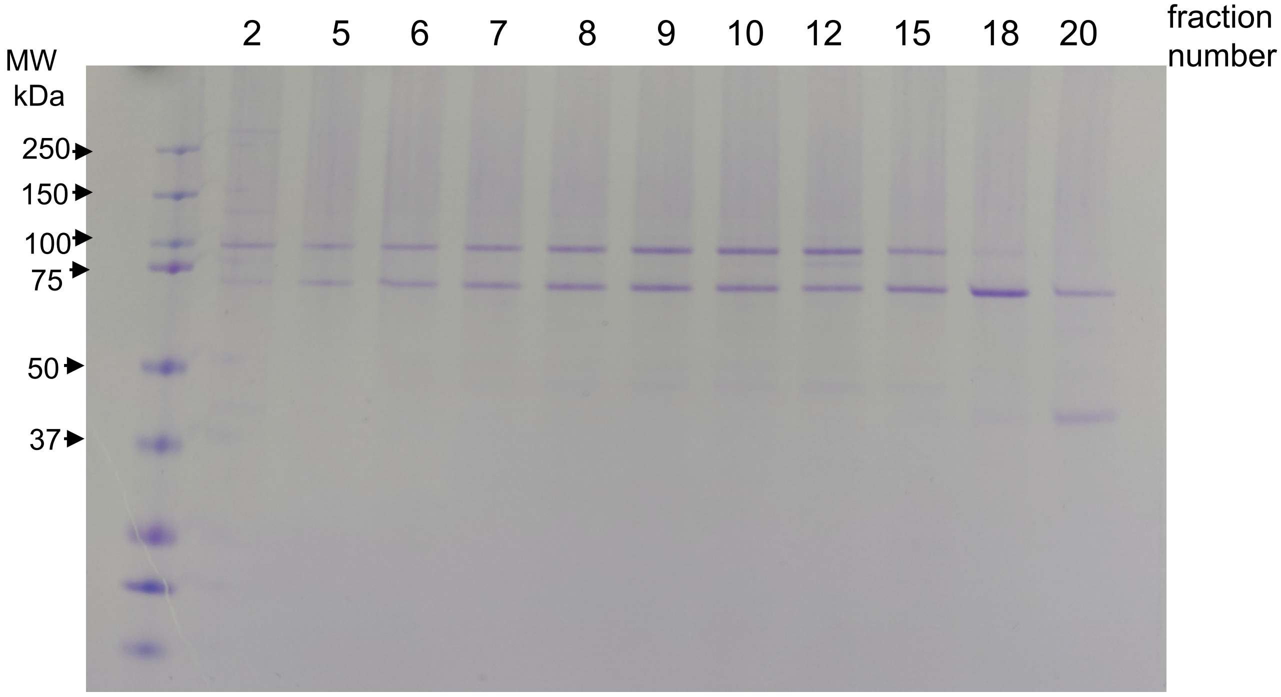

Suppl. Fig. 1. Expression of PDE6C in insect cells in the absence of AIPL1.
A. SEC elution profile of PDE6C expressed in Sf9 cell in the absence of AIPL1. Arrows indicate elution volumes for MW standards.
B. Coomassie-stained gel after SDS-PAGE of SEC fractions.
Note: the spectrum of fraction #2 showed no peak absorbance at 280 or 260, suggesting contains no protein aggregates or DNA.

1 **Acc. #:** Q9U639 **Uniprot ID:** HSP7D_MANSE **Species:** MANSE **Name:** Heat shock 70 kDa protein cognate 4
Organism: Manduca sexta OX=7130 **Existence:** Evidence at transcript level **Version:** 1
Protein MW: 71432.3 **Protein pI:** 5.3 **Protein Length:** 652 **Index:** 186265

```
1   MAKAPAVGID LGTTYSCVGV FQHGKVEIIA NDQGNRTTPS YVAFTDTDRL IGDAAKNQVA MNPNNTIFDA KRLIGRKFED
81  ATVQADMKHW PFEVVSDGGK PKIKVAYKGE DKTFFPEEVS SMVLTKMKET AEAYLGKTVQ NAVITVPAYF NDSQRQATKD
161 AGTISGLNVL RIINEPTAAA IAYGLDKKGS GERNVLIFDL GGGTFDVSIL TIEDGIFEVK STAGDTHLGG EDFDNRMVNH
241 FVQEFKRKYK KDLTTNKRAL RRLRTACERA KRTLSSSTQA SIEIDSLFEG IDFYTSITRA RFEELNADLF RSTMEPVEKS
321 LRDAKMDKSQ IHDIVLVGGS TRIPKVQKLL QDFFNGKELN KSINPDEAVA YGAAVQAAIL HGDKSEEVQD LLLLDVTPLS
401 LGIETAGGVM TTLIKRNTTI PTKQTQTFTT YSDNQPGVLI QVFEGERAMT KDNNLLGKFE LTGIPPAPRG VPQIEVTFDI
481 DANGILNVSA VEKSTNKENK ITITNDKGRL SKEEIERMVN EAEKYRNEDE KQKETIQAKN ALESYCFNMK STMEDEKLKD
561 KISDSDKQTI LDKCNDTIKW LDSNQLADKE EYEHKQKELE GICNPIITKL YQGAGGMPGG MPGGMPGFPG GAPGAGGAAP
641 GGGAGPTIEE VD
```

Num Unique	% Cov	Best Disc Score	Best Expect Val
69	74.7	5.11	7.3e-9

Suppl. Fig. 2. Mass spec identification of the 70 kDa band from the SEC fractions of PDE6C expressed in the absence of AIPL1. The 70 kDa band is identified by Protein prospector as insect HSP70. Note: the *Manduca sexta* HSP70 *sequence from* the database searched by Protein prospector is 99.2% identical to the Spodoptera frugiperda HSP70 sequence.

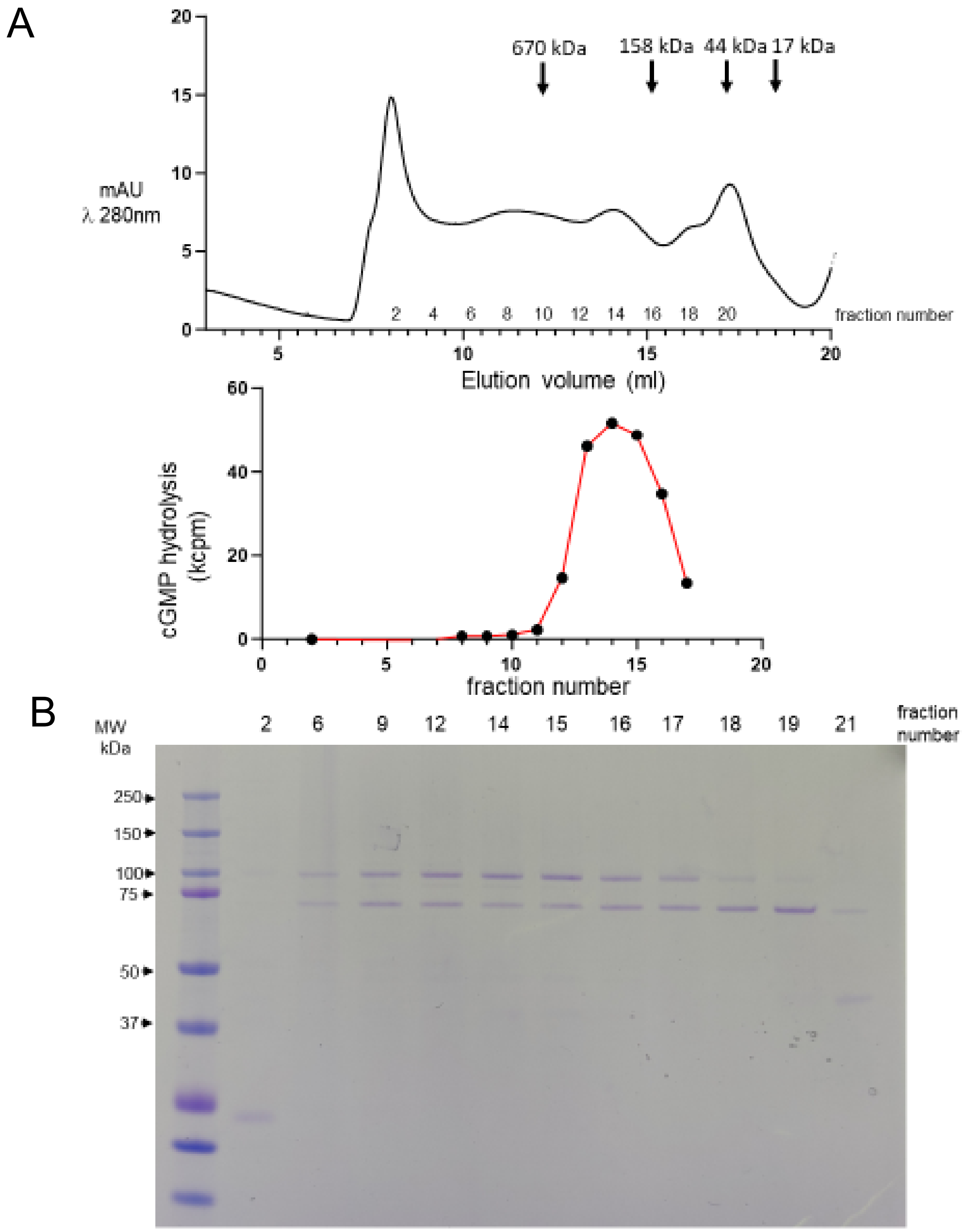

Suppl. Fig. 3. Expression of PDE6C in insect cells in the presence of AIPL1. **A**. SEC elution and activity profiles of PDE6C expressed in Sf9 cell in the presence of AIPL1. **B**. Coomassie-stained gel after SDS-PAGE of SEC fractions. Note that the peak activity corresponds to the PDE6C protein peak.

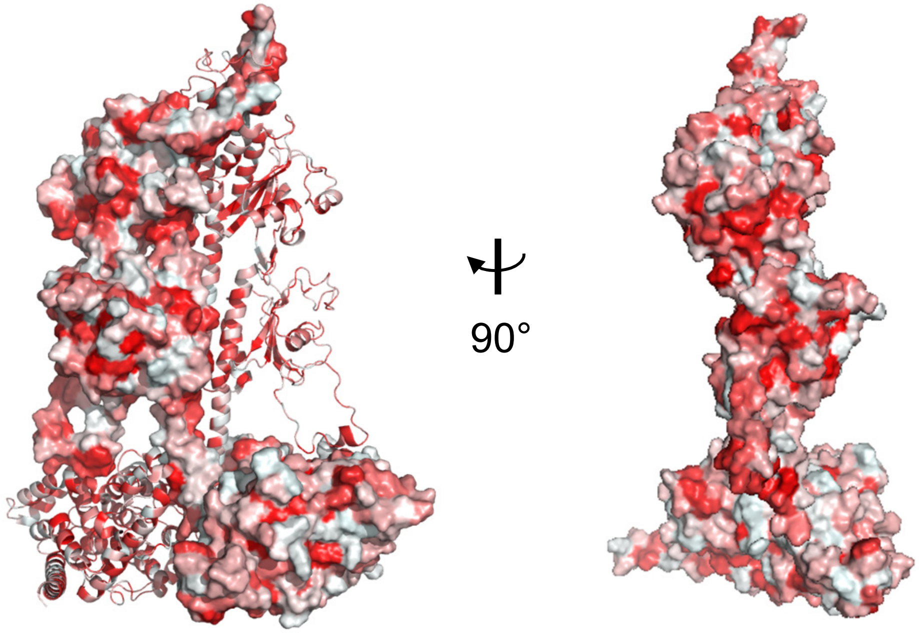

Suppl. Fig. 4. Hydrophobic dimerization interface of the PDE6 catalytic subunits.

The PDEAB dimer (PDB 6MZB) is colored by hydrophobicity (red -hydrophobic, white - hydrophilic). PDE6A is shown as surface and PDE6B is shown as cartoon. Left – PDE6A is extracted from the dimer and rotated to show the dimerization interface.

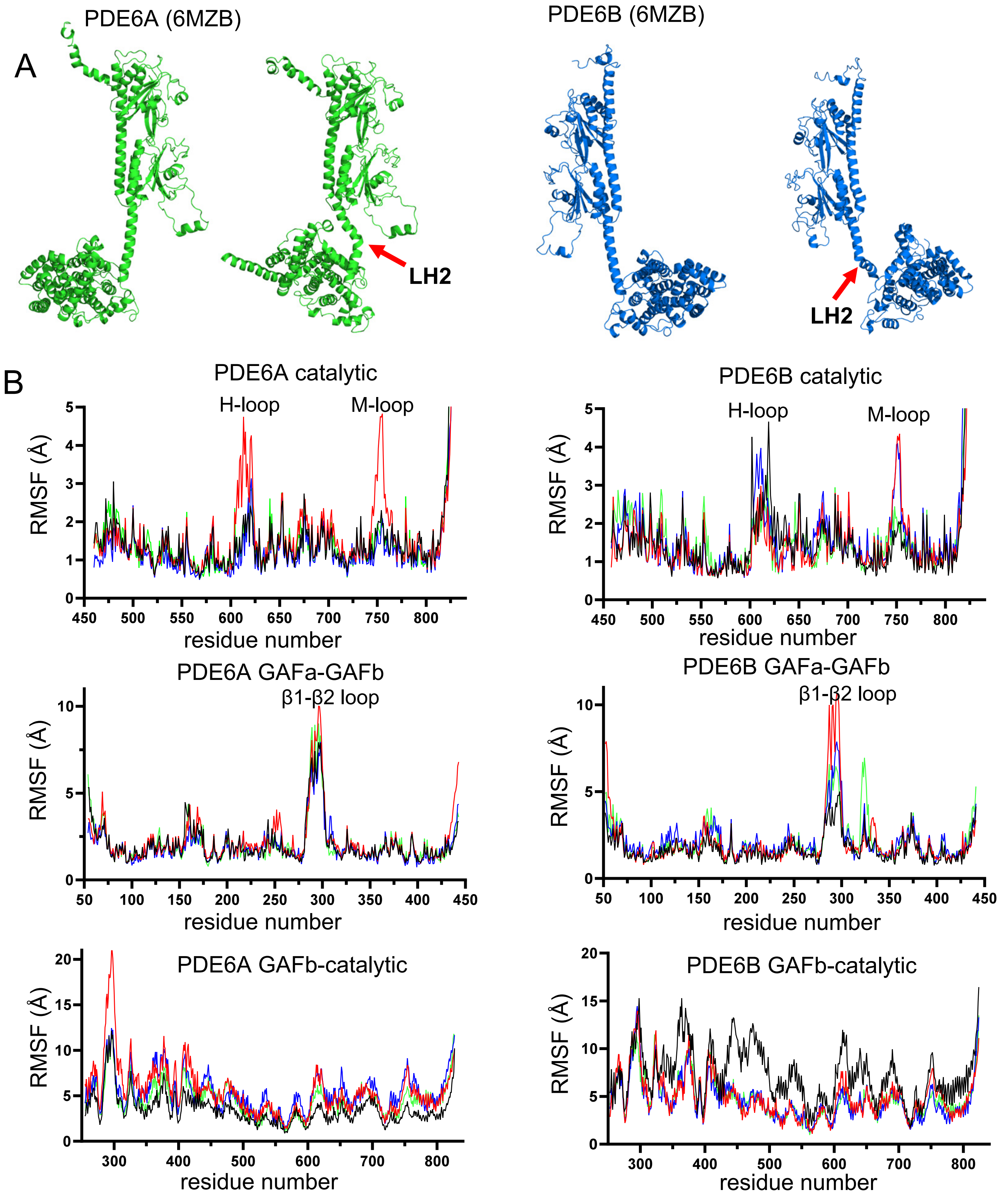

Suppl. Fig. 5. MD simulations of monomeric PDE6 subunits.
A. Starting conformations and representative conformations of PDE6A and PDE6B subunits following MD simulations are shown. Arrows indicate kinked (bended) helix LH2 linking GAFb and the catalytic domains. **B**. RMSF analyses of MD trajectories of PDE6A or PDE6B (4 runs each) where the PDE6 subunits were superposed over the catalytic domain or the GAFa-GAFb region indicate relative stability of these domains except for the flexible H, M loops and the C-termini in the catalytic domain, and the GAFb β1-β2 loop in the GAFa-GAFb region. Increased RMSF values for the superposed GAFb-catalytic domain region reflect relative movements of these domains due to bending of LH2.

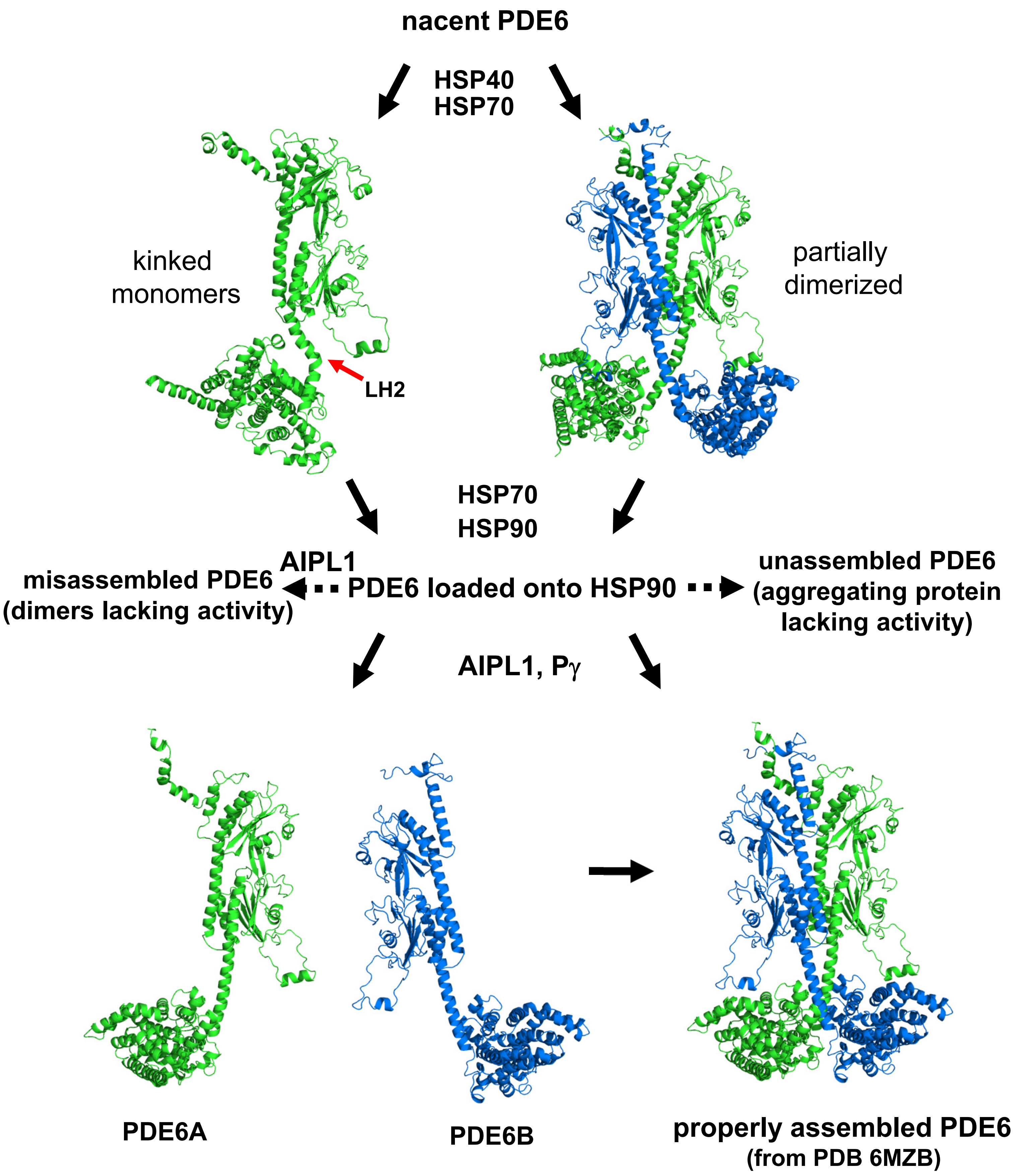

Suppl. Fig. 6. A diagram of potential conformational changes of the PDE6AB subunits during maturation based on MD simulations.
